# A porcine model for pathomorphological age assessment of surgically excised skin wounds

**DOI:** 10.1186/s13028-018-0387-3

**Published:** 2018-05-30

**Authors:** Kristiane Barington, Kristine Dich-Jørgensen, Henrik Elvang Jensen

**Affiliations:** Department of Veterinary and Animal Sciences, Faculty of Health and Medical Sciences, University of Copenhagen, Ridebanevej 3, 1870 Frederiksberg C, Denmark

**Keywords:** Age of wounds, Experimental animal model, Pig, Skin, Ulceration, Veterinary forensic pathology

## Abstract

A porcine model of second intention wound healing was established and gross and histological changes needed for accurate assessing the age of wounds were determined. Twenty-five pigs were anesthetized before incision of four wounds on each. The wounds were left to heal from 1 h to 35 days when the pigs were euthanized. In 14 pigs, biopsies were sampled from two wounds between days 2 and 18. By histological evaluation the following characteristics were found useful for determining the age of wounds: neutrophil:macrophage ratio, angiogenesis, hyperplasia of fibroblasts, presence of hemosiderophages and granulation tissue. The latter was present from day 4 (n = 8 wounds, 100%), but by gross evaluation it was not recognized until day 5 (n = 4 wounds, 100%). From day 4 to 10, the thickness of granulation tissue increased by 1.2 ± 2.4 mm/day. The thickness of collagen fibers within granulation tissue increased throughout the study period, and complete epithelization was reached from day 18.

**Electronic supplementary material:**

The online version of this article (10.1186/s13028-018-0387-3) contains supplementary material, which is available to authorized users.

## Findings

Age assessment of skin wounds in pigs is often requested in veterinary forensic pathology [1, 2]. Wounds in pigs are primarily caused by external trauma, and the age of wounds submitted for forensic investigation ranges from hours and up to several months [3]. Methods to determine the age of wounds have been investigated in porcine models [4–6]. However, in previous studies and unlike wounds in veterinary forensic cases, these wounds were treated with a epinephrine solution to obtain hemostasis or bandaged during healing [4–6]. The aim of the present study was to identify hallmarks for forensic age assessment of porcine wounds by assessing characteristics in experimental wounds during healing by second intention.

Twenty-five specific pathogen free (SPF) female Yorkshire-Landrace crossbred pigs with body weights of 23–34 kg were acclimatized for 1 week, housed individually with bedding of straw and sawdust (Spanvall, Denmark) and numbered 1–25 in the order they arrived to the facility (Table 1).

**Table 1:** Study overview

Wound age	Pig	No. of wounds biopsied	No. of wounds sampled post-mortem	Histological features relevant for age determination	Average neutrophil score + SD	Median neutrophil score (range)	Average macrophage score + SD	Median macrophage score (range)	N:M ratio
1 h	1		4	Clot	4.3 ± 1.7	4.5 (2–6)	1 ± 0	1 (1–1)	4:1
3 h	2, 3		8		5.9 ± 0.4	6 (5–6)	1.5 ± 0.5	1.5 (1–2)	4:1
6 h	4, 5		8		4.8 ± 0.9	5 (3–6)	1.8 ± 0.7	2 (3)	3:1
12 h	6, 7		8	Migration of epithelial cells	5.1 ± 0.6	5 (4–6)	1.6 ± 0.5	2 (1–2)	3:1
1 day	8, 9		8		5.4 ± 1.2	6 (3–6)	2.9 ± 1.0	2.5 (2–4)	2:1
2 days	10–13	4	8	Angiogenesis	4.8 ± 1.6	6 (2–6)	2.3 ± 0.5	2 (2–3)	2:1
3 days	14, 15	4		Fibroblasts	3.8 ± 0.5	4 (3–4)	3.5 ± 0.6	3.5 (3–4)	1:1
4 days	12, 13, 16, 17	4	4	Granulation tissue	3 ± 1.4	3 (1–5)	2.9 ± 0.6	3 (2–4)	1:1
5 days	18,19	4			2 ± 0.8	2 (1–3)	3.3 ± 0.5	3 (3–4)	1:2
6 days	14, 15		4		2.3 ± 1.0	2.5 (1–3)	3.3 ± 0.5	3 (3–4)	1:1
7 days	20, 21	4			1.8 ± 0.5	2 (1–2)	3.8 ± 0.5	4 (3–4)	1:2
8 days	16, 17		4	Hemosiderophages	2.8 ± 1.0	2.5 (2–4)	3.8 ± 0.5	4 (3–4)	1:1
10 days	18, 19		4		2.8 ± 2.1	2.5 (1–5)	3.8 ± 1.0	3.5 (3–5)	1:1
14 days	22, 23	4			1 ± 0	1 (1–1)	2 ± 0	2 (2–2)	1:2
18 days	20, 21, 24, 25	4	4	Complete epithelization	1.1 ± 1.0	1 (0–3)	2 ± 0.5	2 (1–3)	1:2
27 days	22, 23		4		0	0	2 ± 0	2 (2–2)	–
35 days	24, 25		4		0	0	1.5 ± 0.6	1.5 (1–2)	–

Study overview: age of wounds; pigs; number of wounds (sampled by biopsy or post-mortem); first appearance of histological features relevant for age determination; average score ± standard deviation (SD) of neutrophils and macrophages; median score and range of neutrophils and macrophages and the neutrophil:macrophage (N:M) ratio

Infiltration of neutrophils and macrophages was scored as: (0) absent, (1) < 10, (2) 10–20, (3) 21–50, (4) 51–100, (5) 101–200, (6) > 200 using a 40× objective and 10× ocular with FN 22 mm in one high power field (HPF) of 0.237 mm^2^ [9]. The scoring was carried out in the HPF with the highest number of neutrophils and macrophages within areas 1–4 (Fig. 2a, b). From each wound, only the highest score (regardless of area) was registered. At each time point, the average neutrophil and macrophage score, the standard deviation, the median and the neutrophil:macrophage ratio were calculated

*N* neutrophils, *M* macrophages

**Fig. 1:**
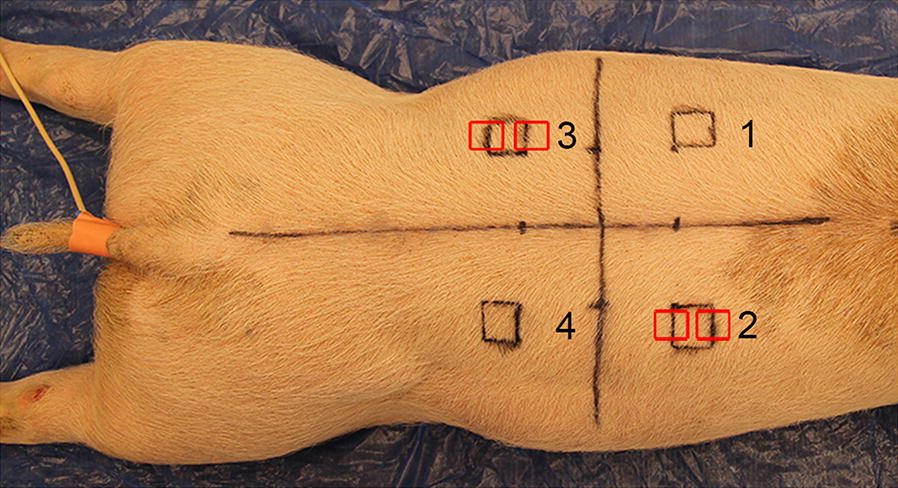
Location of wounds 1–4, experimental pig 21. Each wound was located 4 cm lateral to the spine and 4 cm cranial or caudal to the last rib, respectively. Biopsies were taken from two wound edges at locations 2 and 3 (red boxes) while the pigs were anesthetized. Wounds at location 1 and 4 were sampled post mortem

Each pig was anesthetized (Additional file 1), treated with continuous intravascular infusion of fentanyl (Fentanyl 50 µg/mL, Fentanyl-Hameln; Hameln Pharmaceuticals gmbh, Hameln, Germany), placed in sternal recumbency and prepared for sterile surgery. Four surgical areas of 2 × 2 cm (locations 1–4) were drawn on the back in the paravertebral area (Fig. 1). By incision, full thickness wounds (from the epidermis down to and including the subcutis) were established by removal of 4 cm^2^ skin and left to heal by second intention. Pigs 1–5 were kept anesthetized for 1–6 h and then euthanized, while pigs 6–25 were transported to their pens to wake up (Table 1). At 8 h intervals, pigs were given four injections of buprenorphine (Temgesic, 0.3 mg/mL, Schering-Plough, NJ, USA). None of the pigs showed sign of pain as no increase in respiratory rate, depression, reduced feed intake or mobility were observed. At different time points, pigs 12–25 were anesthetized (Additional file 2) before two full thickness biopsies from each of the wound margins of locations 2 and 3 were sampled by incision (Fig. 1 and Table 1). After 4 h, these pigs received an intramuscular injection of 0.1 mg/kg buprenorphine. The pigs were euthanized from 1 h to 35 days after the creation of the wounds with an overdose of intravenous pentobarbital (Glostrup Apotek, Glostrup, Denmark) (Table 1).

Following euthanasia, cross sections of the wounds that had not previously been biopsied were sampled for histology. In total, 100 wounds were evaluated, i.e. 28 wounds were biopsied and 72 wounds were sampled after euthanasia (Table 1). Granulation tissue was defined as a red and hemorrhagic connective tissue filling the wound bed and recorded as present or absent by gross inspection [7]. In wounds that were biopsied, evaluations were only recorded until the day of biopsy sampling.

**Fig. 2:**
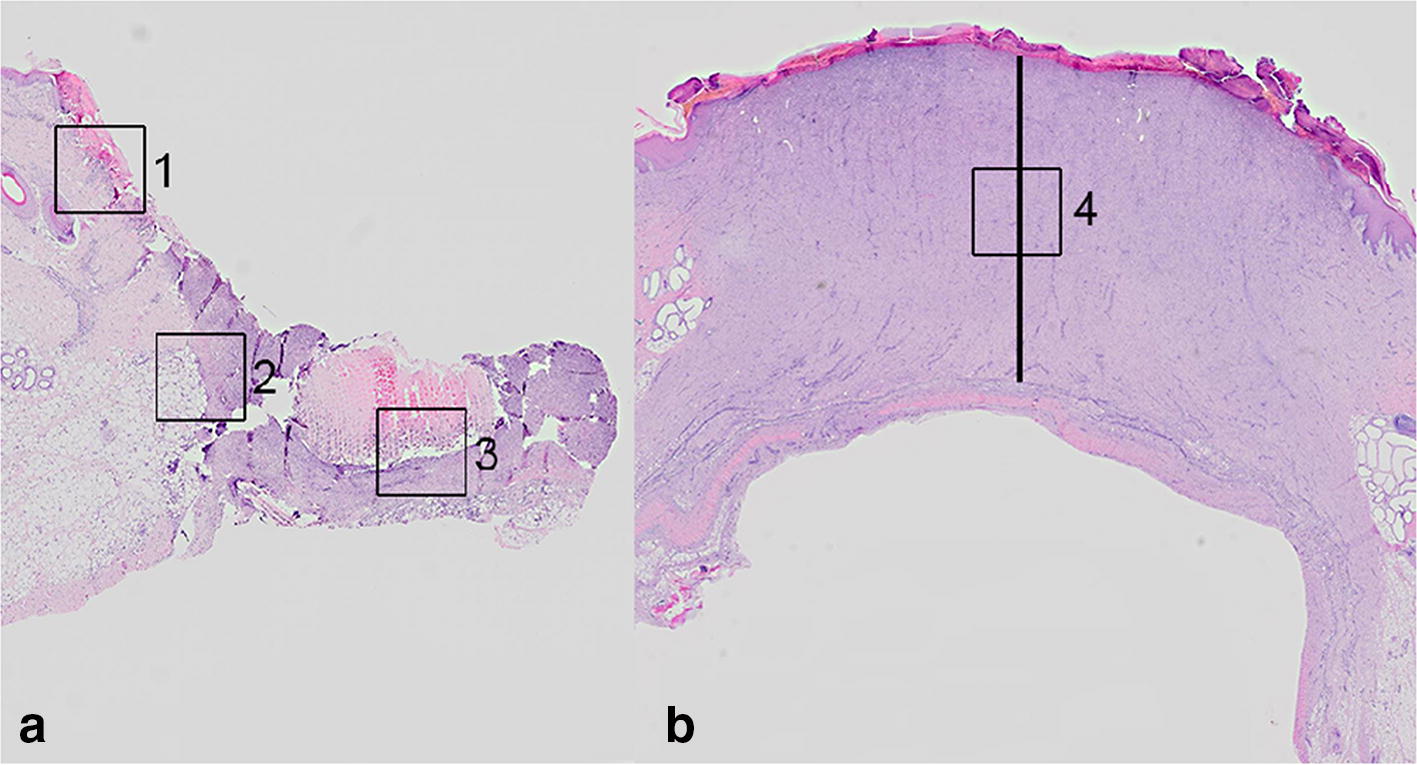
Areas subjected to histological evaluation of biopsies (**a**) or cross sections of wounds (**b**), hematoxylin and eosin stain. **a** Histological evaluation of wounds aged 1 h to 10 days was carried out in areas 1–3 (3 mm × 3 mm). Area 1: at the wound edge, dermis. Area 2: at the wound edge, subcutis. Area 3: central in the wound bed. **b** Histological evaluation of wounds aged 14 to 35 days was carried out in area 4 (3 mm × 3 mm), i.e. central in the granulation tissue. The thickness of the granulation tissue was measured at the center of the wound (straight line)

Histological preparation of the tissue was done as previously described [8]. Sections stained with hematoxylin and eosin were evaluated in four areas (3 × 3 mm) depending on the age of the wounds and therefore not blinded (Fig. 2a, b). All registrations were carried out by a single veterinary pathologist. Infiltration of neutrophils and macrophages was scored as: (0) absent, (1) < 10; (2) 10–20; (3) 21–50; (4) 51–100; (5) 101–200; (6) > 200 using a 40 × objective and 10× ocular with FN 22 mm in one high power field (HPF) of 0.237 mm^2^ [9]. The scoring was carried out in the HPF with the highest number of neutrophils and macrophages within areas 1–4 (Fig. 2a, b). From each wound, only the highest score (regardless of area) was registered, i.e. one score per wound. Similarly, when two biopsies were sampled from one wound both biopsies were evaluated, but only the highest score was registered. At each time point, the average neutrophil and macrophage score, the standard deviation (SD), the median and the neutrophil:macrophage ratio were calculated. Hemorrhage, hyperplasia of fibroblasts and endothelial cells and angiogenesis were registered as present or absent in areas 1–4 (Figs. 2a, b). Granulation tissue was defined as fibroblasts and collagen arranged perpendicular to the new proliferating vessels (angiogenesis) [7]. When present, the thickness of granulation tissue was measured at the center of the wound from the top of the wound bed (not including epidermis) to the level of normal tissue components using the software Delta Pix 2.3.5 (Smorum, Denmark) (Fig. 2b). Differences in the thickness of granulation tissue, due to the location of the wound (location 1–4), were evaluated by comparing locations 1 and 4 and locations 2 and 3 using the paired *t* test (P < 0.05). Moreover, at each time point the mean thickness of granulation tissue and SD were calculated (Additional file 3).

Epithelization was characterized as: (0) absent, (1) hyperplasia of epithelial basal cells, (2) migration of epithelial cells, and (3) full epithelization.

Randomly selected sections from all time points were stained with Perl’s Prussian blue, Masson’s trichrome, and Picrosirius red in order to confirm the presence of hemosiderophages and visualization of collagen in the wound bed [10, 11]. The change in polarization color from green to yellow to red in the aging wounds stained with Picrosirius red is due to increasing thickness of collagen [11]. Immunohistochemistry based on polyclonal rabbit anti-human von Willebrand Factor antibody (A0082, Agilent, USA) was used to confirm the presence of vessels [12].

**Fig. 3:**
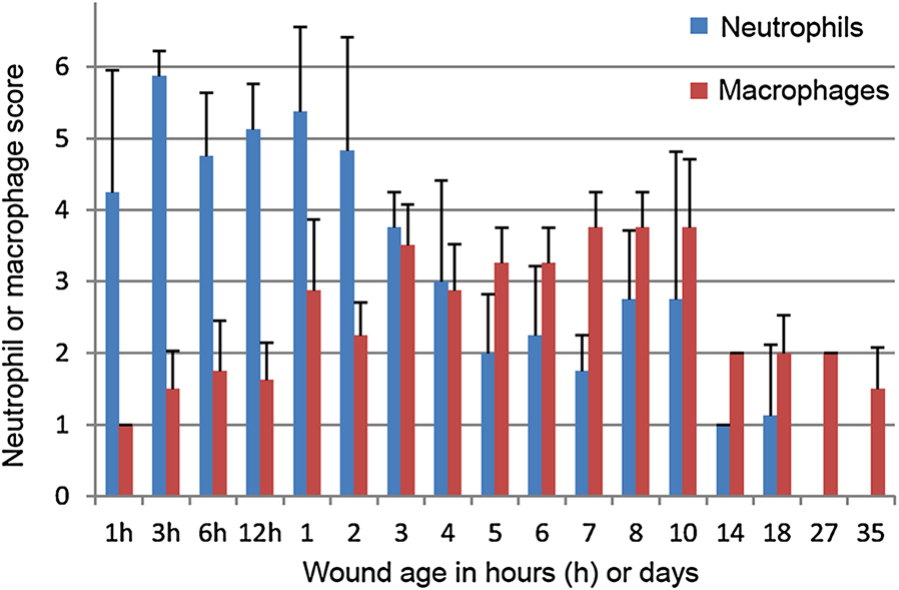
The average score ± standard deviation of neutrophils and macrophages in wounds being from 1 h to 35 days

The first appearance of the most important histological manifestations for determining the age of wounds is presented in Table 1. Hemostasis was present within 1 h where a clot had formed in the wound bed. Infiltration of neutrophils around vessels and in the dermal and subcutaneous tissue was seen from 1 h to day 18 (90 of 92 wounds, 98%). In comparison, a intravital reaction (i.e. active infiltration of leukocytes) is not found in porcine wounds inflicted postmortem or seconds before death [8, 13]. The number of neutrophils peaked after 3 h to 2 days with a score greater than 4.5 (Fig. 3) (Additional file 4).

Infiltration of macrophages in the dermis and subcutis was seen in the wounds (n = 100 wounds, 100%) throughout the experimental period but peaked from day 7 to 10 with a score greater than 3.5 (Fig. 3) (Additional file 4). In accordance with this, macrophages peaked at day 7 in wounds in a previous porcine excisional wound model [14]. From 8 to 35 days, hemosiderophages were present. In comparison, hemosiderophages, which to the authors knowledge have not been noticed in porcine wounds hitherto, have been reported in human wounds already at day 3 and appeared regularly from day 8 [15].

Based on the neutrophil:macrophage ratio, wounds could roughly be assessed as being from 1 to 3 h, 6 to 12 h, 1 to 2 days or more than 3 days (Table 1). Contamination of the wounds with bedding material was inevitable and may have affected the inflammatory reaction [16]. However, in forensic cases, wounds are also subjected to infection, foreign bodies and continuous trauma, which can affect the neutrophil:macrophage ratio [16]. Hyperplasia of endothelial cells, angiogenesis and hyperplasia of fibroblast were observed in all wounds from 12 h, 2 and 3 days, respectively. Our observations are in accordance with previous records on experimental wounds in pigs, in which hyperplasia of fibroblasts was seen within 3 days [17]. Moreover, in forensic porcine wounds, we have previously predicted angiogenesis and hyperplasia of fibroblasts to begin at 16 h to 3 days [3].

**Fig. 4:**
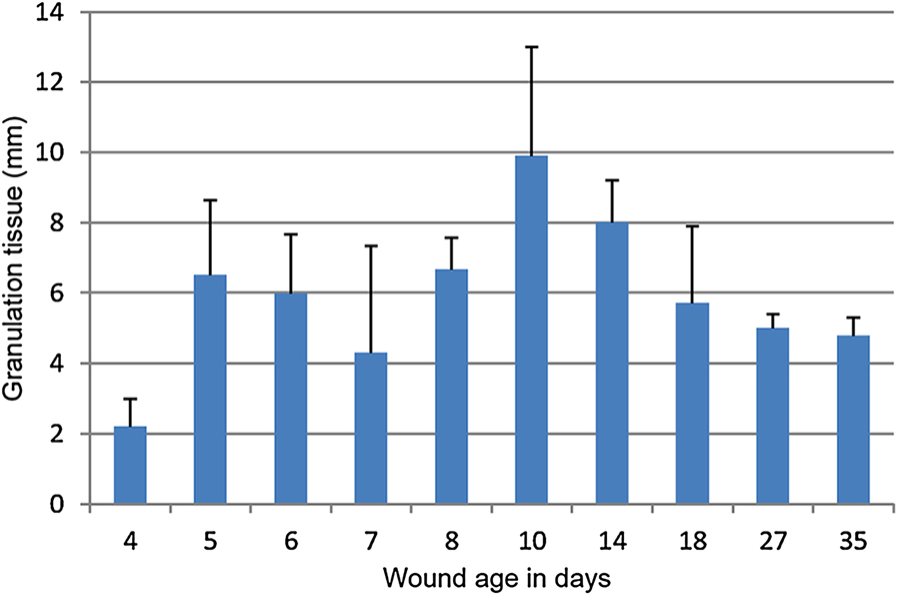
The average thickness ± standard deviation of granulation tissue in wounds being from 4 to 35 days

Histologically, granulation tissue was present at day 4 (n = 8 wounds, 100%), but at gross evaluation it was not recognized until day 5 (n = 4 wounds, 100%). Granulation tissue thickness on days 4 to 7 varied from approximately 2–6 mm and increased to nearly 10 mm on day 10 (growth rate: 1.2 ± 2.4 mm per day). However, from day 10 to 35 the thickness decreased by 0.3 ± 0.3 mm per day (Fig. 4) (Additional file 3). In forensic cases of porcine wounds, granulation tissue with thicknesses of 5, 15 and 27 mm have been used to estimate wound age as being 4–7 days, 8–28 days and > 28 days, respectively [3]. The decrease in granulation tissue thickness seen in the experimental wounds but apparently not in forensic cases is likely due to the initial depth of the lesion, accompanying infection, sequestration of necrotic tissue and sustained injury [16]. Therefore, the growth rate of granulation tissue up to 10 days should probably be seen as the maximum by which it can be formed. The wide SD of the average growth rate is likely due to inter pig variation as no differences in granulation tissue thickness between locations 1 and 4 (P = 0.26) and locations 2 and 3 (P = 0.07) were found.

**Fig. 5:**
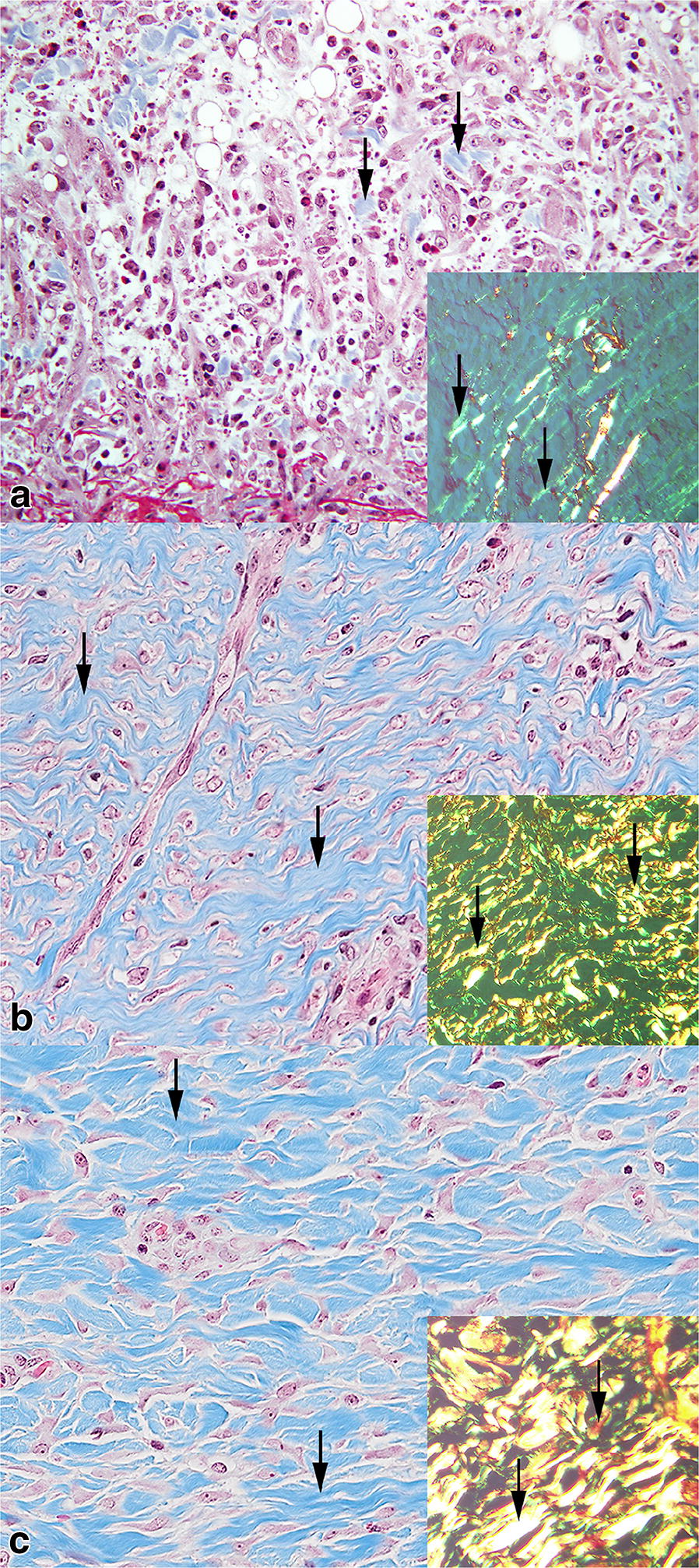
Porcine wound beds being 4 days (**a**), 10 days (**b**) and 27 days (**c**) old. **a** A scarce amount of blue stained collagen is present (arrows), Masson’s trichrome stain. Inset: Green collagen fibers are visible under polarized light (arrows), Picrosirius red stain. **b** Massive amounts of blue stained collagen are present (arrows), Masson’s trichrome stain. Inset: Green, yellow and few red collagen fibers are visible in polarized light (arrows), Picrosirius red stain. **c** Massive amounts of blue stained collagen are present (arrows), Masson’s trichrome stain. Inset: Yellow, red and a few green collagen fibers are visible in polarized light (arrows), Picrosirius red stain

Masson’s trichrome and Picrosirius red stains both confirmed the presence of newly formed collagen from day 4 (Fig. 5a). Gradually, more collagen was deposited throughout the experimental period (Figs. 5a–c).

Hyperplasia and early migration of epithelial basal cells were seen from 12 h (2/8 wounds, 25%) and complete epithelization was present in wounds being from 18 to 35 days old. This is in agreement with another porcine model based on 2 × 2 cm full thickness wounds, in which complete epithelization was achieved on day 28 [18].

**Fig. 6:**
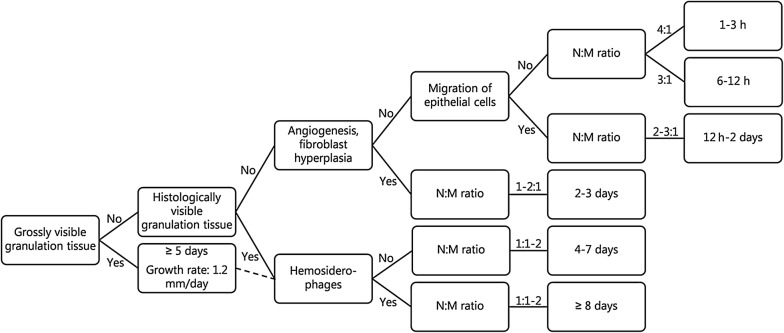
Decision diagram for determining the age of wounds based on gross and histological evaluation

Histological features of significant importance for assessing the age of porcine wounds were identified (Table 1, Fig. 6). Age assessment of wounds without granulation tissue should be based on the ratio between neutrophils and macrophages and the presence of angiogenesis and fibroblast hyperplasia (Fig. 6). In wounds containing granulation tissue, the thickness of it was useful for wound age determination and the neutrophil:macrophage ratio, deposition of collagen and presence of hemosiderophages can further support the assessment (Fig. 6).

## Additional files

**Additional file 1.** Sedation and general anesthesia for surgical incision of wounds.

**Additional file 2.** Anesthesia and reversal of anesthesia for sampling of biopsies.

**Additional file 3.** Granulation tissue was measured at the center of the wound from the top of the wound bed (not including epidermis) and excluding underlying fat tissue and the fibrous fascia of the muscle.

**Additional file 4.** Infiltration of neutrophils and macrophages was scored as: (0) absent; (1) < 10; (2) 10–20; (3) 21–50; (4) 51–100; (5) 101–200; (6) > 200 using a 40× objective and 10× ocular with FN 22 mm in one high power field (HPF) of 0.237 mm^2^. The scoring was carried out in the HPF with the highest number of neutrophils and macrophages within areas 1–4 in the wounds (Fig. 2a, b). From each wound, only the highest score was registered, i.e. one neutrophil score and one macrophage score per wound. Location of the wound (1–4), wound age, neutrophil score and macrophage score are presented.

## Abbreviations

SPF: specific pathogen free, i.e. free from infection with *Mycoplasma hyopneumoniae*, *Actinobacillus pleuropneumoniae serotype* 1–10 and 12, Porcine Reproductive Respiratory Syndrome, *Brachyspira hyodysenteriae*, *Pasteurella multocida*, *Sarcoptes Scabiei* var*. Suis*. and *Haematopinus suis*
