# Supplementary material for: A porcine model for pathomorphological age assessment of surgically excised skin wounds
Source: Acta Vet Scand. 2018 May 30;60:33. doi: 10.1186/s13028-018-0387-3 (PMC5977753; doi:10.1186/s13028-018-0387-3)
Supplement: Supplementary file 1 — Additional file 1. Sedation and general anesthesia for surgical incision of wounds. [file 13028_2018_387_MOESM1_ESM.docx]

**Additional file 1:** Sedation and general anesthesia for surgical incision of wounds

| **Effect** | **Drugs** | **Dose** | **Route of administration** | **Trade name and manufacturer** |
| --- | --- | --- | --- | --- |
| Sedation | Mixture of tiletamin, zolazepam,xylazin,  ketamine and butorphanol | 0.1 mL/kg | Intramuscular injection | Zoletil 50 Vet. 125 mg, Virbac, Animal Health, Carros, France (without solvent); Xysol vet. 20 mg/mL, ScanVet Animal Health A/S, Fredensborg, Denmark (6.25 mL); Ketaminol Vet 100 mg/mL, Intervet International BV, Holland (1.25 mL); Torbugesic Vet 10 mg/mL, ScanVet Animal Health A/S, Fredensborg, Denmark (2.5 mL) |
| Anesthesia | Propofol | To effect | Intravenous infusion | Propofol 10 mg/mL, B Braun, Melsungen, Germany |
