# Supplementary material for: A porcine model for pathomorphological age assessment of surgically excised skin wounds
Source: Acta Vet Scand. 2018 May 30;60:33. doi: 10.1186/s13028-018-0387-3 (PMC5977753; doi:10.1186/s13028-018-0387-3)
Supplement: Supplementary file 2 — Additional file 2. Anesthesia and reversal of anesthesia for sampling of biopsies. [file 13028_2018_387_MOESM2_ESM.docx]

**Additional file 2:** Anesthesia and reversal of anesthesia for sampling of biopsies

| **Effect** | **Drugs** | **Dose** | **Route of administration** | **Trade name and manufacturer** |
| --- | --- | --- | --- | --- |
| Anesthesia | A mixture of ketamine,  dexmedetomidin  and butorphanol | 10 mg/kg  40 µg/kg  0.2 mg/kg | Intramuscular injection | Ketaminol Vet 100 mg/mL, Intervet International BV, Holland (1.25 mL); Dexdomitor, 0.5 mg/mL, Orion Corporation, Orion Pharma, Finland; Torbugesic Vet 10 mg/mL, ScanVet Animal Health A/S, Fredensborg, Denmark (2.5 mL) |
| Reversal of anesthesia | Atipamezole hydrochloride | 20 µg/kg | Intramuscular injection | Antisedan 5.0 mg/mL, Orion Corporation, Orion Pharma, Finland |
