# Supplementary material for: A porcine model for pathomorphological age assessment of surgically excised skin wounds
Source: Acta Vet Scand. 2018 May 30;60:33. doi: 10.1186/s13028-018-0387-3 (PMC5977753; doi:10.1186/s13028-018-0387-3)
Supplement: Supplementary file 3 — Additional file 3. Granulation tissue was measured at the center of the wound from the top of the wound bed (not including epidermis) and excluding underlying fat tissue and the fibrous fascia of the muscle. [file 13028_2018_387_MOESM3_ESM.docx]

**Additional file 3:** Granulation tissue was measured at the center of the wound from the top of the wound bed (not including epidermis) and excluding underlying fat tissue and the fibrous fascia of the muscle.

|  | **Granulation tissue thickness (mm) in wounds** | | | |
| --- | --- | --- | --- | --- |
| **Wound age in days** | **Location 1** | **Location 2** | **Location 3** | **Location 4** |
| 4 | 0,9 | 2,6 | 2,5 | 1,7 |
| 4 | 1,7 | 3,4 | 2,6 | 2,4 |
| 5 | n/a | 7,9 | 5,5 | n/a |
| 5 | n/a | 8,6 | 4,0 | n/a |
| 6 | 5,8 | n/a | n/a | 4,2 |
| 6 | 8,7 | n/a | n/a | 5,3 |
| 7 | n/a | 6,7 | 7,2 | n/a |
| 7 | n/a | 1,5 | 1,9 | n/a |
| 8 | 6,4 | n/a | n/a | 7,8 |
| 8 | 5,7 | n/a | n/a | 6,7 |
| 10 | 10,9 | n/a | n/a | 8,1 |
| 10 | 13,8 | n/a | n/a | 6,9 |
| 14 | n/a | 9,3 | 7,5 | n/a |
| 14 | n/a | 8,4 | 6,6 | n/a |
| 18 | 5,8 | n/a | n/a | 3,9 |
| 18 | 2,2 | n/a | n/a | 4,1 |
| 18 | n/a | 7,9 | 8,7 | n/a |
| 18 | n/a | 6,8 | 6,0 | n/a |
| 27 | 5,0 | n/a | n/a | 4,8 |
| 27 | 4,4 | n/a | n/a | 5,7 |
| 35 | 5,1 | n/a | n/a | 5,2 |
| 35 | 4,9 | n/a | n/a | 4,0 |

n/a=not applicable
