# Supplementary material for: A porcine model for pathomorphological age assessment of surgically excised skin wounds
Source: Acta Vet Scand. 2018 May 30;60:33. doi: 10.1186/s13028-018-0387-3 (PMC5977753; doi:10.1186/s13028-018-0387-3)
Supplement: Supplementary file 4 — Additional file 4. Infiltration of neutrophils and macrophages was scored as: (0) absent; (1) < 10; (2) 10–20; (3) 21–50; (4) 51–100; (5) 101–200; (6) > 200 using a 40× objective and 10× ocular with FN 22 mm in one high power field (HPF) of 0.237 mm2. The scoring was carried out in the HPF with the highest number of neutrophils and macrophages within areas 1–4 in the wounds (Fig. 2a, b). From each wound, only the highest score was registered, i.e. one neutrophil score and one macrophage score per wound. Location of the wound (1–4), wound age, neutrophil score and macrophage score are presented. [file 13028_2018_387_MOESM4_ESM.docx]

**Additional file 4:** Infiltration of neutrophils and macrophages was scored as: 0) absent; 1) < 10; 2) 10 to 20; 3) 21 to 50; 4) 51 to 100; 5) 101 to 200; 6) > 200 using a 40x objective and 10x ocular with FN 22 mm in one high power field (HPF) of 0.237 mm^2^. The scoring was carried out in the HPF with the highest number of neutrophils and macrophages within areas 1 to 4 of the wounds (Figs. 2A and B). From each wound, only the highest score was registered, i.e. one neutrophil score and one macrophage score per wound. In the table beneath pig, location (1-4), wound age, neutrophil score and macrophage score are presented.

| **Pig** | **Location** | **Wound age** | **Neutrophils (Score 0-6)** | **Macrophages (Score 0-6)** |
| --- | --- | --- | --- | --- |
| 0 | 1 | 1 hour | 5 | 1 |
| 0 | 2 | 1 hour | 6 | 1 |
| 0 | 3 | 1 hour | 2 | 1 |
| 0 | 4 | 1 hour | 4 | 1 |
| 1 | 1 | 3 hours | 6 | 1 |
| 1 | 2 | 3 hours | 6 | 1 |
| 1 | 3 | 3 hours | 6 | 2 |
| 1 | 4 | 3 hours | 6 | 1 |
| 2 | 1 | 3 hours | 6 | 1 |
| 2 | 2 | 3 hours | 6 | 2 |
| 2 | 3 | 3 hours | 6 | 2 |
| 2 | 4 | 3 hours | 5 | 2 |
| 3 | 1 | 6 hours | 6 | 1 |
| 3 | 2 | 6 hours | 5 | 1 |
| 3 | 3 | 6 hours | 4 | 1 |
| 3 | 4 | 6 hours | 3 | 2 |
| 4 | 1 | 6 hours | 5 | 2 |
| 4 | 2 | 6 hours | 5 | 3 |
| 4 | 3 | 6 hours | 5 | 2 |
| 4 | 4 | 6 hours | 5 | 2 |
| 5 | 1 | 12 hours | 5 | 1 |
| 5 | 2 | 12 hours | 5 | 2 |
| 5 | 3 | 12 hours | 5 | 2 |
| 5 | 4 | 12 hours | 4 | 2 |
| 6 | 1 | 12 hours | 6 | 1 |
| 6 | 2 | 12 hours | 5 | 1 |
| 6 | 3 | 12 hours | 6 | 2 |
| 6 | 4 | 12 hours | 5 | 2 |
| 7 | 1 | 1 day | 4 | 4 |
| 7 | 2 | 1 day | 6 | 3 |
| 7 | 3 | 1 day | 6 | 4 |
| 7 | 4 | 1 day | 3 | 4 |
| 8 | 1 | 1 day | 6 | 2 |
| 8 | 2 | 1 day | 6 | 2 |
| 8 | 3 | 1 day | 6 | 2 |
| 8 | 4 | 1 day | 6 | 2 |
| 11 | 2 | 2 days | 2 | 3 |
| 11 | 3 | 2 days | 2 | 3 |
| 12 | 2 | 2 days | 4 | 2 |
| 12 | 3 | 2 days | 4 | 2 |
| 9 | 1 | 2 days | 6 | 3 |
| 9 | 2 | 2 days | 6 | 2 |
| 9 | 3 | 2 days | 6 | 2 |
| 9 | 4 | 2 days | 6 | 2 |
| 10 | 1 | 2 days | 4 | 2 |
| 10 | 2 | 2 days | 6 | 2 |
| 10 | 3 | 2 days | 6 | 2 |
| 10 | 4 | 2 days | 6 | 2 |
| 13 | 2 | 3 days | 4 | 3 |
| 13 | 3 | 3 days | 4 | 3 |
| 14 | 2 | 3 days | 3 | 4 |
| 14 | 3 | 3 days | 4 | 4 |
| 15 | 2 | 4 days | 5 | 2 |
| 15 | 3 | 4 days | 3 | 4 |
| 16 | 2 | 4 days | 1 | 2 |
| 16 | 3 | 4 days | 1 | 3 |
| 11 | 1 | 4 days | 4 | 3 |
| 11 | 4 | 4 days | 4 | 3 |
| 12 | 1 | 4 days | 3 | 3 |
| 12 | 4 | 4 days | 3 | 3 |
| 17 | 2 | 5 days | 2 | 3 |
| 17 | 3 | 5 days | 1 | 3 |
| 18 | 2 | 5 days | 3 | 4 |
| 18 | 3 | 5 days | 2 | 3 |
| 13 | 1 | 6 days | 2 | 3 |
| 13 | 4 | 6 days | 1 | 3 |
| 14 | 1 | 6 days | 3 | 4 |
| 14 | 4 | 6 days | 3 | 3 |
| 19 | 2 | 7 days | 2 | 4 |
| 19 | 3 | 7 days | 2 | 4 |
| 20 | 2 | 7 days | 2 | 3 |
| 20 | 3 | 7 days | 1 | 4 |
| 15 | 1 | 8 days | 4 | 4 |
| 15 | 4 | 8 days | 2 | 3 |
| 16 | 1 | 8 days | 2 | 4 |
| 16 | 4 | 8 days | 3 | 4 |
| 17 | 1 | 10 days | 1 | 3 |
| 17 | 4 | 10 days | 1 | 3 |
| 18 | 1 | 10 days | 5 | 5 |
| 18 | 4 | 10 days | 4 | 4 |
| 21 | 2 | 14 days | 1 | 2 |
| 21 | 3 | 14 days | 1 | 2 |
| 22 | 2 | 14 days | 1 | 2 |
| 22 | 3 | 14 days | 1 | 2 |
| 23 | 2 | 18 days | 1 | 2 |
| 23 | 3 | 18 days | 1 | 2 |
| 24 | 2 | 18 days | 3 | 1 |
| 24 | 3 | 18 days | 1 | 2 |
| 19 | 1 | 18 days | 2 | 3 |
| 19 | 4 | 18 days | 0 | 2 |
| 20 | 1 | 18 days | 1 | 2 |
| 20 | 4 | 18 days | 0 | 2 |
| 21 | 1 | 27 days | 0 | 2 |
| 21 | 4 | 27 days | 0 | 2 |
| 22 | 1 | 27 days | 0 | 2 |
| 22 | 4 | 27 days | 0 | 2 |
| 23 | 1 | 35 days | 0 | 1 |
| 23 | 4 | 35 days | 0 | 2 |
| 24 | 1 | 35 days | 0 | 2 |
| 24 | 4 | 35 days | 0 | 1 |
